# Advantage of First-Line Therapeutic Drug Monitoring-Driven Use of Infliximab for Treating Acute Intestinal and Liver GVHD in Children: A Prospective, Single-Center Study

**DOI:** 10.3390/cancers15143605

**Published:** 2023-07-13

**Authors:** Natalia Maximova, Daniela Nisticò, Guglielmo Riccio, Alessandra Maestro, Egidio Barbi, Barbara Faganel Kotnik, Annalisa Marcuzzi, Erika Rimondi, Antonello Di Paolo

**Affiliations:** 1 Department of Pediatrics, Institute for Maternal and Child Health—IRCCS Burlo Garofolo, 34137 Trieste, Italy; natalia.maximova@burlo.trieste.it (N.M.); egidio.barbi@burlo.trieste.it (E.B.); 2 Department of Medical, Surgical and Health Sciences, University of Trieste, 34127 Trieste, Italy; 3 Pharmacy and Clinical Pharmacology Unit, Institute for Maternal and Child Health—IRCCS Burlo Garofolo, 34137 Trieste, Italy; 4 Department of Hematology and Oncology, University Children’s Hospital, 1000 Ljubljana, Slovenia; barbara.faganel@gmail.com; 5 Department of Translational Medicine, University of Ferrara, 44121 Ferrara, Italy; 6 Department of Translational Medicine and LTTA Centre, University of Ferrara, 44121 Ferrara, Italy; 7 Department of Clinical and Experimental Medicine, University of Pisa, 56126 Pisa, Italy; antonello.dipaolo@med.unipi.it

**Keywords:** acute graft-versus-host disease, children, TNF-α, infliximab, therapeutic drug monitoring

## Abstract

**Simple Summary:**

Acute GVHD is a common life-threatening complication of allogeneic HSCT and the second most common cause of death in allogeneic HSCT recipients following relapse of the primary disease. Despite advances in GVHD therapy, many patients do not respond sufficiently, and biomarkers to predict treatment success are not established. This issue also concerns the TNF-α blocker infliximab. This single-center prospective observational study was designed to assess the effectiveness of first-line infliximab treatment compared to infliximab use in second- or further-line therapy in pediatric allogeneic HSCT recipients. Our data show that using first-line, TDM-driven infliximab to treat aGVHD in children may result in better clinical outcomes and good tolerability, with a variable pattern of serum cytokines and drug clearance according to the timing of treatment and disease extension, respectively.

**Abstract:**

The high serum concentrations of TNF-α characterize acute graft-versus-host disease (aGVHD), for which infliximab treatment may be beneficial. In 28 pediatric patients, four doses of 10 mg/kg infliximab every seven days were administered after steroid failure (Standard Group, n = 14) or as a first-line therapy (Early Group, n = 14). Population pharmacokinetic analyses and evaluation of serum cytokines were performed. After two months of treatment, complete response in gastrointestinal and liver aGVHD was achieved in 43% and 100% of patients in the Standard and Early groups, respectively. During follow-up, four patients in the Standard Group (but none in the Early Group) experienced an aGVHD recurrence. Viral infections occurred more frequently in the Standard Group after the fifth dose. Infliximab clearance did not differ between groups or according to treatment outcome for each organ involved in aGVHD, whereas serum levels of cytokines significantly differed. Therefore, present findings show that use of first-line, TDM-driven infliximab to treat aGVHD in children may result in better clinical outcomes and tolerability, with a different pattern of cytokines generated according to the moment of beginning of treatment.

## 1. Introduction

Acute graft-versus-host disease (aGVHD) is a common life-threatening complication of allogeneic hematopoietic stem cell transplantation (alloHSCT), which is distinguished based on systemic inflammation that mostly attacks the liver, skin, and gut, which occurs in 25 to 50% of patients. aGVHD is the second most common cause of death in alloHSCT recipients folllowing relapse of the primary disease [1,2]. aGVHD frequency has decreased over time in matched related and unrelated donor transplantations. Furthermore, the introduction of post-transplant cyclophosphamide in the context of haploidentical transplantation has been a radical turn, allowing doctors to perform mismatched transplants safely and effectively, including from related donors. Despite this development, the absolute number of patients experiencing this complication has increased due to the growing number of alloHSCT procedures performed worldwide [3,4]. Corticosteroids are the basis of the first-line treatment for aGVHD, producing sustained responses in less than 50% of patients [5]. Currently, there is no commonly accepted treatment for patients with steroid-refractory (SR) aGVHD, and their long-term survival is significantly poor, irrespective of the type of secondary therapy used [6,7].

Cytokines play a crucial role in the pathogenesis of aGVHD. The inflammatory milieu created by pre-transplant conditioning generates, in response, many pro-inflammatory cytokines. Interleukin 1 (IL-1), tumor necrosis factor-alpha (TNF-α), interferon-gamma (IFN-γ), and interleukin 6 (IL-6), which originate from both conditioning-induced tissue damage and donor T-cells, have been recognized as the main mediators of aGVHD in experimental models [8].

It was documented that TNF-α > 100 pg/mL levels, in the first three months after alloHSCT, strongly correlated with aGVHD, as well as veno-occlusive disease (VOD), endothelial leakage syndrome, and interstitial pneumonitis [9,10]. Knowledge of the etiologic role of TNF-α in the pathogenesis of GVHD guided the interest in use of infliximab for treating GVHD [11]. Inhibition of TNF-α has been suggested in all phases of aGVHD treatment, as prevention, a part of primary treatment, and, most commonly, a treatment for steroid-refractory or steroid-dependent aGVHD [12]. 

In our Institute, infliximab has been used to treat aGVHD as a second- or further-line therapy since 2000. In 2018, we introduced infliximab therapeutic drug monitoring (TDM) and began using infliximab as a part of the first-line therapy to treat intestinal and liver manifestations of aGVHD. This single-center prospective observational study was designed to assess the effectiveness of first-line infliximab treatment compared to infliximab use in second- or further-line therapy in pediatric alloHSCT recipients. Further, the study aims to investigate whether the drop in infliximab plasma concentrations could be associated with clinical response and the production of pro- and anti-inflammatory cytokines.

## 2. Materials and Methods

### 2.1. Study Design and Population

This prospective single-center observational study was conducted at the Pediatric Onco-Hematology Department and Bone Marrow Transplant Center of the Institute for Maternal and Child Health—IRCCS Burlo Garofolo, Trieste, Italy, from 2018 to 2022. The Institutional Review Board of the IRCCS Burlo Garofolo (reference no. IRB RC 18/22) approved the protocol, and the study was conducted following the guidelines of the Declaration of Helsinki (Clinicaltrials.gov code: NCT05362630). The patients’ parents gave their written consent for us to collect and use personal data for research purposes. From June 2018 to July 2022, consecutive pediatric patients with hematological malignancies and hematological non-malignant diseases who underwent infliximab treatment for aGVHD after allogeneic HSCT were included in this study. All patients underwent proactive infliximab TDM with anti-drug antibodies detection. In addition, 27 pro- and anti-inflammatory cytokines’ blood levels were measured at baseline and, subsequently, for every infliximab TDM. Furthermore, the patients’ demographic, clinical, and laboratory data were recorded and anonymized.

### 2.2. Definitions and Endpoints

All patients underwent alloHSCT and were treated according to standard myeloablative protocols based on chemotherapy and radiation therapy, as previously described [13]. A myeloablative conditioning regimen was defined as total body irradiation ≥ 8 Gy, busulfan 16 mg/kg, or melphalan 140 mg/m^2^ [14]. GVHD prophylaxis was performed with tacrolimus. Additional GVHD prophylaxis included mycophenolate mofetil for the matched unrelated donor (MUD), with the addition of post-transplant cyclophosphamide in the case of a haploidentical donor. Serotherapy with anti-thymocyte globulin (ATG) was also assessed as an independent variable. As previously described, prevention and treatment of infection and other elements of transplant-specific supportive care were performed according to institutional standard practices [15].

Both classical aGVHD, which occurs within 100 days of HSCT, and persistent or late-onset aGVHD were included. Histological GVHD grading was performed based on a published staging system, and clinical grading was determined according to the criteria for aGVHD [16,17,18]. No response to standard high-dose (1–2 mg/kg) steroid treatment within seven days or progression after three days was defined as steroid-refractory aGVHD, while inability to taper steroid dose after the initial response was defined as steroid-dependent aGVHD. Evaluation of response was performed according to previously described diagnostic criteria for aGVHD [17,18]. Treatment responses were categorized as complete response (CR), partial response (PR), non-response, or progression. A CR to infliximab was defined as the absence of symptoms related to aGVHD. A PR was defined as the improvement in at least one stage in the severity of aGVHD in one organ without deterioration in any other organ; the response had to last for at least three weeks. Non-response or progression was defined by the absence of improvement in aGVHD, deterioration of aGVHD in any organ by at least one stage, the development of aGVHD manifestations in a previously unaffected organ, and the use of any additional agents to control the disease.

### 2.3. Infliximab Administration and TDM

All patients who gave written informed consent to off-label infliximab use received infliximab biosimilars developed by Inflectra^®^ (Hospira, Zagreb, Croatia) or Flixabi^®^ (Samsung Bioepsis, Delft, The Netherlands) at the standard dose of 10 mg/kg/dose intravenously in two-hour infusions on a weekly basis, with an initial plan for four doses. Infliximab treatment in second- or further-line therapy for steroid-refractory or steroid-dependent aGVHD was defined as standard infliximab use. Infliximab treatment started together with or in the first three days of steroid treatment as part of first-line treatment was defined as early infliximab use. The decision regarding the timing of the start of infliximab treatment was made by the physician.

The infliximab serum trough levels were measured immediately before the next drug infusion. In case of multiple administrations, the clearance of infliximab after a single dose or after the last dose was determined via weekly TDM for in-patients and every hospital visit for out-patients.

Infliximab trough levels were quantitatively determined using RIDA^®^QUICK IFX Monitoring test and the read out was performed on the RIDA^®^QUICK SCAN II version 2.1 (R-Biopharm AG, Darmstadt, Germany). ADAs were measured via enzyme-linked immunosorbent assays (ELISA) when infliximab plasma levels were less than or equal to 1.5 μg/mL. Patients with ADA above ten units per milliliter (AU/mL) were considered positive.

### 2.4. Analysis of Cytokines and Chemokines

The analysis of 27 cytokines and chemokines, namely IL-1β, IL-1ra, IL-2, IL-4, IL-5, IL-6, IL-7, IL-8, IL-9, IL-10, IL-12(p70), IL-13, IL-15, IL-17, Eotaxin, FGF basic, G-CSF, GM-CSF, IFN-γ, IP-10, MCP-1 (MCAF), MIP-1α, PDGF-bb, MIP-1β, RANTES (CCL5), TNF-α, and VEGF, was carried out on plasma samples via multiple immunoassays and using a bead-based magnetic sensor (27 human-Bio-Plex assays) (BIO-RAD Laboratories, Milan, Italy) following the manufacturer’s instructions. The plasma samples analyzed were extracted from patients treated with both standard and early use of infliximab (Supplementary Table S1), as well as from pediatric patients hospitalized for elective surgical interventions or within the scope of elective diagnostic procedures, who were used as a control (Supplementary Table S2). Data were acquired via a Bio-Plex 200 reader, and a digital processor and Bio-Plex Manager^®^ 6.0 software converted data into median fluorescence intensity and concentration (pg/mL).

Plasma concentrations of infliximab (expressed as nmol/L) were analyzed using the non-linear mixed-effect modeling software Monolix version 2020R1 (Lixoft, Antony, France), adopting the SAEM algorithm. The model development was guided by both numerical and graphical outputs generated via Monolix software (Monolix Suite 2021R2), including a significant variation in the objective function value (OFV) of ≥3.84 and ≥6.83 units in the forward inclusion and backward exclusion phases, respectively. In addition, estimated values of pharmacokinetic parameters, covariance matrices, and relative standard error values (RSE%), together with graphical outputs, were carefully considered. In particular, observed concentrations versus population and individual predictions, individual-weighted residual (IWRES) distributions and correlations with times after dose and observed values, and prediction-corrected visual predictive checks (pc-VPC) were evaluated.

Model development started with one- and two-compartment models, in which additive, proportional, and combined error models were tested. The interindividual (IIV) and interoccasion (IOV) variabilities were defined via exponential models. As each occasion could last for several weeks, continuous covariates, such as body weight, creatinine clearance, serum albumin, etc., were coded as regressors within the database according to Monolix instructions. Both categorical (i.e., gender) and continuous covariates (i.e., age, body weight, creatinine clearance) with possible effects of drug pharmacokinetics were identified by checking graphical and numerical relationships. They were then introduced into the developing models. In particular, continuous covariates (Cov) were integrated as power models, with individual values (Covi) normalized to the population median value (Covmedian) as follows:

$$\theta_{i}=\theta_{pop}\times {\left(\frac{{Cov}_{i}}{{Cov}_{median}}\right)}^{\beta }$$

where *θ_i_* and *θ_pop_* are the individual and population values of the pharmacokinetic parameter, respectively, and *β* is the allometric exponent (i.e., 0.75 for clearances and 1 for volumes of distribution). As per the general rule, covariates were retained within the model if they significantly improved model performance, according to the criteria listed above.

### 2.5. Statistical Analysis

An organ-specific response to infliximab was described as a proportion of patients with any residual disease using the Kaplan–Meier model. Differences between study groups were analyzed using the log-rank method. Stratification of patients was also performed to distinguish between grades of response to therapy. The drug’s safety was assessed by describing the number of cases of infection in both study groups and the proportion of patients who developed infections at different doses of infliximab. Statistical analysis was not performed for stratification or safety assessment due to the limited number of enrolled patients. Values were expressed as mean ± standard deviation values or as median and minimum–maximum ranges depending on the context and parameters. All statistics and graphs were obtained using the R Studio software (R version 4.2.0).

## 3. Results

### 3.1. Patient and Disease Characteristics

Twenty-eight pediatric patients were treated with off-label use of infliximab biosimilars after an alloHSCT. The first fourteen patients received infliximab for steroid-refractory or steroid-dependent aGVHD (Standard Group), and the remaining fourteen patients used infliximab as part of first-line therapy (Early Group). Patients’ demographic, transplant, and clinical characteristics are summarized in Table 1. The most common indication of transplantation was acute leukemia in both the Standard Group and the Early Group (54% and 50%, respectively), followed by non-malignant diseases in the Standard Group (22%) and myelodysplastic syndromes in the Early Group (29%). One patient in the Standard Group and eight in the Early Group received a bone marrow graft (7% vs. 54%), while the remainder received peripheral blood grafts with stem cell sources from HLA-identical sibling (7% vs. 29%), matched unrelated (72% vs. 42%), and haploidentical (21% vs. 29%) donors. There were no umbilical cord blood recipients. All patients received pre-transplant myeloablative conditioning. Primary GVHD prophylaxis was performed with tacrolimus in the Standard and Early Groups in 7% and 29% of patients, respectively; tacrolimus combined with mycophenolate mofetil was used in 72% and 42% of patients, respectively; and tacrolimus combined with mycophenolate mofetil and post-transplant cyclophosphamide was used in 21% and 29% of patients, respectively. 

### 3.2. GVHD and Treatment Response

The median onset of aGVHD was 15 (range, 9 to 220) days in the Standard Group, mainly with a maximum grade of 3 or 4 (10 patients, 72%), and 22.5 (range, 9 to 93) days in the Early Group, mostly with a grade 2 (10 patients, 72%). Sites of involvement included the gastrointestinal system (seven patients (50%) in the Standard Group vs. ten (71.4%) in the Early Group), liver (all patients in the Standard Group vs. five (35.7%) in the Early Group), and skin (nine patients (64.3%) in the Standard Group vs. two (14.3%) in the Early Group).

Initial steroid treatment was started at medians of 1.9 (1 to 3) days in the Standard Group and 1.7 (1 to 4) days in the Early Group. Infliximab was started as a second- or further-line therapy at a median of 24.7 (range, 7 to 51) days after the initiation of steroids, with the median number of infliximab administrations being 4.7 (range, 2 to 10), and as a part of a first-line therapy at a median of 1.6 (range, 0 to 3) days, with the median number of infusions being 1.5 (range, 1 to 3).

Two patients (7%) in the Standard Group died during infliximab treatment. Both deaths were not directly attributable to infliximab therapy: one patient died of VOD, and another patient died of non-infectious diffuse alveolar hemorrhage. 

In the Standard Group, two months after the initiation of treatment, 12 patients (86%) responded to infliximab therapy (n = 6, 43% with CR, and n = 6, 43% with PR). Organ-specific responses were 14% (n = 1 CR, n = 1 PR), 36% (n = 2 CR, n = 3 PR), and 86% (n = 2 CR, n = 10 PR) for skin, gastrointestinal, and liver involvement, respectively. Seven patients (50%) with cutaneous, three patients (21%) with gastrointestinal, and 2 patients (7%) with liver involvement had progression of aGVHD or required further treatment. Four patients (29%) received additional infliximab therapy for a GVHD recurrence.

**Figure 1:**
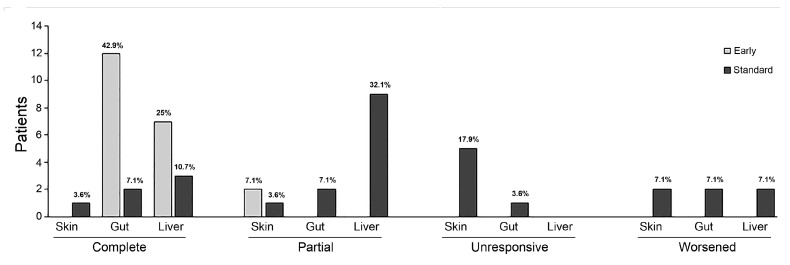
Organ-specific responses at two months after initiation of infliximab therapy show a better response in the intestinal and liver involvements in both groups. In contrast, skin involvement demonstrated poor response to both early and standard administration. The graphs represent each percentage among whole population.

In the Early Group, two months after the initiation of treatment, 14 patients (100%) with gastrointestinal and liver aGVHD obtained CR, whereas two patients (7%) with skin involvement obtained PR. Organ-specific responses at two months in both groups are shown in Figure 1. Nine patients (64%) achieved CR after only one dose. None of the patients in the Early Group experienced aGVHD recurrence during the follow-up.

### 3.3. Adverse Effects and Infections

**Figure 2:**
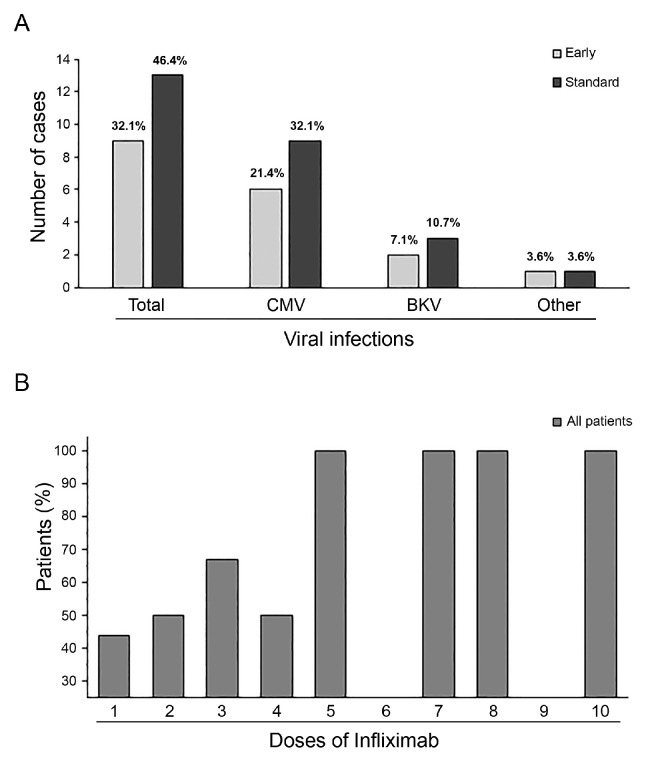
Overall incidence of virus reactivation and distribution by virus type in both groups (graph represents each percentage among whole population) (**A**). Increase in viral reactivation events with a rise in infliximab administrations (**B**).

We did not observe adverse reactions during infliximab infusions. All patients received antimicrobial, antifungal, and antivirus prophylaxis. No bacterial or fungal infections or deaths due to infections were recorded during the follow-up period. Despite taking antivirus prophylaxis during infliximab treatment, viral reactivation or infections were observed in 17 patients (60.7%), with 10 cases (71.4%) in the Standard Group and 7 cases (50%) in the Early Group. Five patients (17.8%) had contemporary reactivation of two or more viruses (three patients in the Standard Group and two in the Early Group). Cytomegalovirus was the most frequent reactivation that required treatment in both groups, followed by the BK virus. In addition, one patient in the Standard Group had Epstein–Barr virus reactivation, and one patient in the Early Group had Adenovirus disease (Figure 2A). Furthermore, the percentage of viral reactivation or infection significantly increased in the Standard Group after the fifth infliximab dose, as shown in Figure 2B. 

### 3.4. Infliximab Pharmacokinetic and Cytokines Evaluations

The database included twenty-eight patients, eleven and six of whom had two or three occasions, respectively. Two hundred and forty-seven observations were available (mean and minimum-maximum range, 4.7 and 1–10 values per occasion). A one-compartment structural model with first-order elimination and a proportional model error adequately fitted the observations. The IIV was sequentially included for both CL and V parameters (decreases in OFV of −431.497 and −59.853 units, respectively, from the initial model), while IOV was added to CL (OFV, −108.084 units). For both pharmacokinetic parameters, patients’ body weight was the only covariate included, as allometric scaling led to a significant improvement in model performance (OFV, −63.359 units). Equations used in the model were written as follows:

$${CL}_{i}=0.146\times {\left(\frac{{WGT}_{i}}{26.2}\right)}^{0.75}$$

$$V_{i}=3.431\times {\left(\frac{{WGT}_{i}}{26.2}\right)}^{1}$$

where WGTi is the individual body weight of each patient.

**Figure 3:**
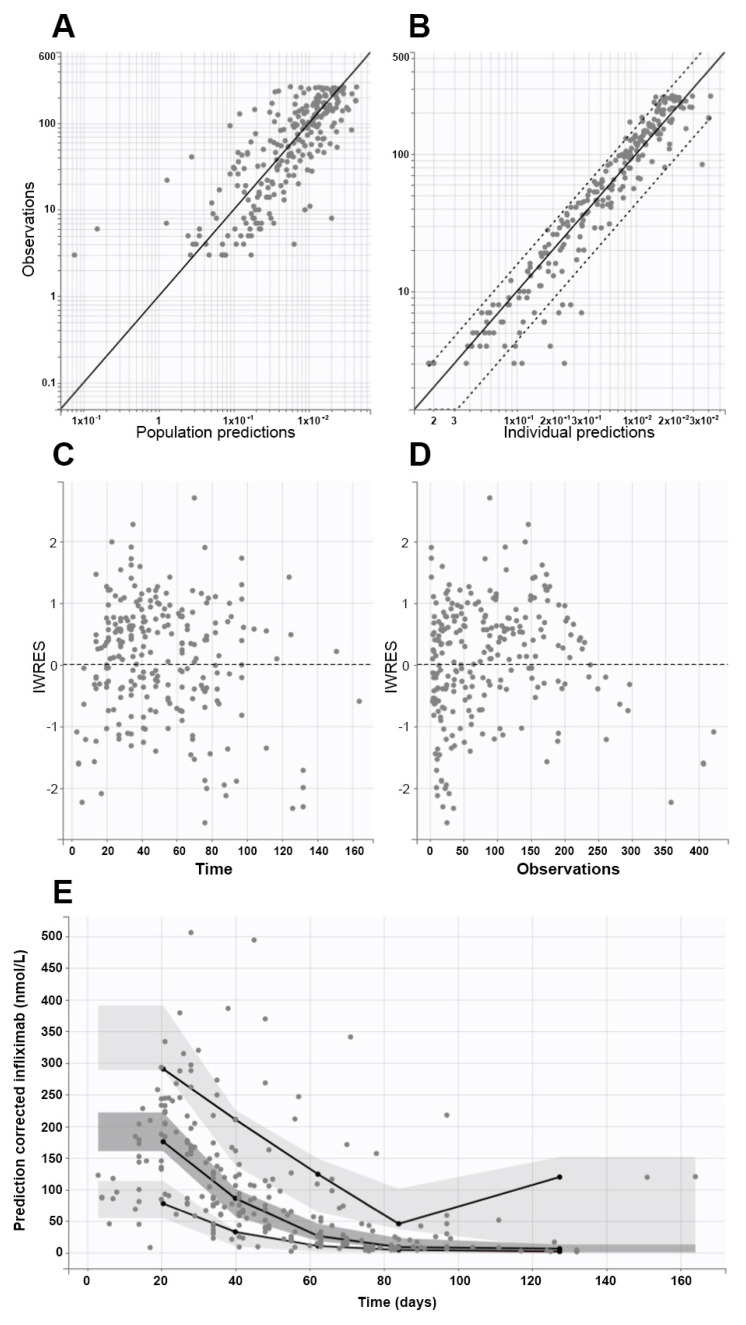
Goodness-of-fit plots from the final model, which show population (**A**) and individual (**B**) prediction values plotted against observed values (continuous and dashed black lines represent the identity lines and 90% confidence interval limits, respectively), individual weighted residuals (IWRES) versus times (**C**), and IWRES versus observations (**D**). Plot (**E**) shows the prediction-corrected visual predictive check (pcVPC) plot (continuous lines are the 5th, 50th, and 95th empirical percentiles, while light and dark grey areas represent 95% confidence interval limits of the empirical percentiles). In all graphs, closed circles represent observed data.

Findings of fixed and random effects are presented in Table 2, while diagnostic goodness-of-fit plots and pcVPC showed the good prediction capabilities of the final model (Figure 3). 

On the first occasion, patients had CL and V values equal to 0.163 ± 0.097 L/day and 3.47 ± 1.3 L, respectively, without significant differences according to gender or disease severity at the beginning of infliximab treatment. Furthermore, no significant differences in CL values over time were observed when the CL value on the first occasion was compared to those calculated on the second (0.176 ± 0.079 L/day) and third occasions (0.174 ± 0.053 L/day) in 11 and 6 patients, respectively.

Early or late infliximab administration did not influence CL values (0.169 ± 0.056 and 0.158 ± 0.056 L/day, respectively). Similar results were observed for treatment outcomes in each organ involved in aGVHD. On the contrary, infliximab CL differed significantly based on the extension of the disease, but not in terms of its severity. Indeed, 23 patients with a maximum of two sites of disease had a significantly lower CL (0.138 ± 0.043 L/day) than the remaining 5 individuals with three organs involved (0.202 ± 0.039 L/day, *p* < 0.05).

**Figure 4:**
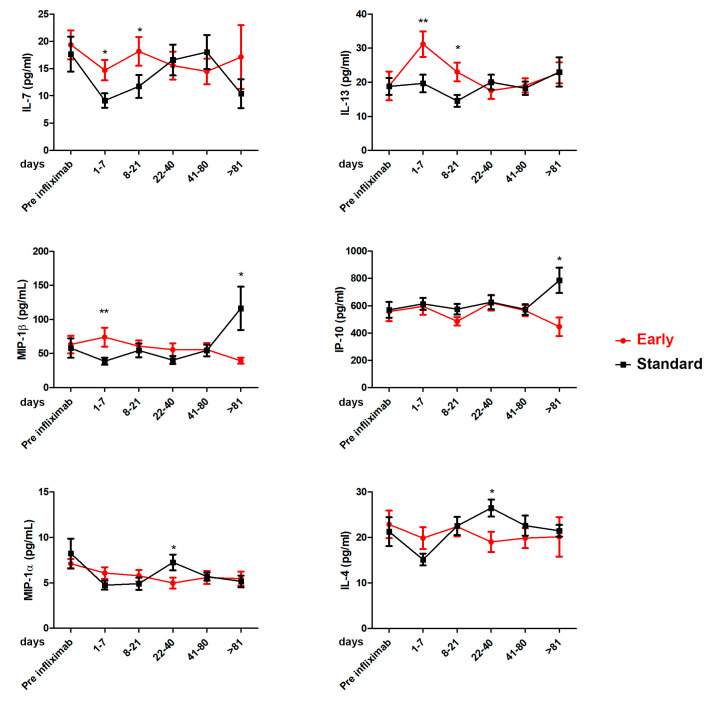
Cytokine profile in Standard and Early groups in sera patients: IL-4, IL-7, IL-13, IP-10, MIP-1α, and MIP-1β. Cytokine levels were analyzed before the treatment with infliximab (pre-infliximab) and then at 1–7, 8–21, 22–40, 41–80, and >81 days after the initiation of treatment. Standard Group vs. Early Group, * *p* < 0.05; ** *p* < 0.001 based on Mann–Whitney test.

The serum samples collected at every therapeutic drug monitoring were analyzed for cytokines levels by comparing the Standard and Early groups (Supplementary Table S1). Figure 4 shows statistically significant differences between the two groups in terms of the IL-7, IL-13, MIP-1β, IP-10, MIP-1a, and IL-4 serum levels found. The early use of infliximab induced higher levels of IL-7 and IL-13 compared to the Standard Group in a period spanning the first day after treatment and day 21 (median of 7 and 8 days for the Standard and the Early Groups, respectively). Conversely, standard infliximab use induced higher serum levels of MIP-1β and IP-10 between 22 and 40 days (median of 34 and 32 days for the Standard and the Early Groups, respectively) and MIP-1a and IL-4, with significantly increased levels after 81 days of treatment.

## 4. Discussion

Pediatric aGVHD differs from adult aGVHD in terms of incidence, severity, and response to treatment [19]. Therapeutic strategies for pediatric SR aGVHD are characterized by high variability in second-line treatment worldwide, and the optimal practice needs to be defined. No prospective studies evaluated which second-line treatment is most effective, and, as a result, there is a lack of standardization in managing pediatric SR aGVHD [20]. Most pediatric centers consider patients to be SR for aGVHD after a shorter period than adult practice. No pediatric data support the recommendation that initiating second-line treatment earlier improves outcomes [19]. A fair number of studies that assessed the efficacy of infliximab in adult patients in both second- [11,12,21,22,23,24,25] and first-line therapy [26,27,28,29,30] have been published, providing conflicting results. Two pediatric studies evaluating the efficacy of infliximab in second-line therapy concluded that infliximab is well tolerated and effective in children with SR aGVHD. However, infection is common, and mortality remains high, as do frequent recurrences [31,32].

To the best of our knowledge, our study is the first study to focus on using infliximab as the first-line TDM-driven therapy for treating aGVHD in children. We observed an excellent response after a single administration in 64% of patients in the Early Group. In contrast, none of the patients in the Standard Group achieved CR after the first administration. Overall complete response to infliximab was 100% in the Early group, compared to 43% in the Standard Group. No recurrences were observed after early infliximab use, compared to almost a third of patients relapsed in the Standard Group.

Our data suggest that the response to infliximab is organ related, hence the better response that we obtained for the intestinal and liver involvements in both groups. In contrast, skin involvement demonstrated poor response to both early and standard administration. The single partial responses obtained are probably attributable to cortisone therapy, rather than to infliximab. 

The current study demonstrated excellent drug tolerance with no infusion-related events occurring. Neither neurologic nor cardiac complications attributed to infliximab were detected in any patient. Infliximab has previously been associated with hepatic injury [33,34]. Our analysis did not observe a worsening of the liver function tests. Only one patient in the Standard Group had a hepatic failure, which was their cause of death. We attributed his hepatic worsening to VOD concurrent with aGVHD. 

TNF-α blockers such as infliximab have been linked to an increased risk of infections, especially opportunistic fungal infections, with reported variable incidences of invasive fungal infection ranging from 6% to 50% [35,36,37,38,39,40,41]. No proven fungal infection, bacterial sepsis, tuberculosis, or atypical mycobacterium were noted in our cohort. Although our virus infection rate was high (61%), these findings are comparable with the historical virus reactivation incidence data obtained by our Center. Furthermore, the Standard Group consisted of patients with advanced-grade aGVHD who receivd aggressive immunosuppressive therapy, particularly with high doses of corticosteroids, making them more susceptible to virus infections.

Our study also demonstrated that the two groups’ expression levels of several cytokines (IL-7, IL-13, IL-4, MIP-1α, MIP-1β, and IP-10) significantly differed.

These results are supported by pre-clinical studies conducted on mouse models that emphasized differentiation and an organ-specific cytokine profile during GVHD [42,43,44,45]. In these preliminary studies, the target organs that seemed to be most affected by cytokines imbalance were the liver, intestine, and skin. These results were mainly associated with increased expression of IL-4 [42,44], MIP-1α [45], and IP-10 [46,47]. In addition, a specific affinity for gastrointestinal tract damage was correlated with a variation in IL-13 levels. In particular, studies conducted on animal models have highlighted the central role of IL-13 in driving permeability in the intestinal epithelium and its secretion mechanisms [48]. Moreover, the roles of IL-7, MIP-1α, and MIP-1 β as biomarkers that predict the onset of GVHD and relapse has been demonstrated, highlighting the role played by the cytokine complex in the pathogenesis of GVHD [49,50,51,52].

The final population pharmacokinetic model fitted observed data with good approximation, as judged by the numerical and graphical results. It is worth noting that mean pharmacokinetic parameter values for CL (0.163 L/day) and V (3.47 L) agreed with those already published for infliximab administered in a pediatric population [53,54,55]. Even the inclusion of patients’ body weights within the model mirrored examples found in the literature [53], despite no further covariates being included in previous models (i.e., serum albumin and erythrocyte sedimentation rate), significantly improved the fitting of measured plasma concentrations available in the present study. Anti-infliximab antibodies were not detected. Hence, their effect on infliximab pharmacokinetics was not evaluated, as occurred in the previous studies [54].

It is worth noting that children with at maximum two organs involved had significantly lower drug CL than patients with a wide extension of the disease. On the contrary, differences in infliximab CL were not associated with disease severity at the beginning of the treatment, did not anticipate the treatment outcome, and did not change during the first three treatment cycles (for which the database had at least six patients for each occasion). Those results further strengthen the important correlation between the onset of therapy, disease control, and cure. Indeed, the earlier the administration of infliximab, the better the clinical outcome of pharmacological treatment achieved in children with aGVHD.

The number of enrolled patients certainly represents a limitation of the study, even if the protocol was the first to investigate the clinical role of TDM-guided infliximab in pediatric aGVHD after HSCT, as well as the first in which the evaluation of drug pharmacokinetics and pharmacodynamics were shown to be useful to investigate potential biomarkers of treatment outcome and tolerability.

## 5. Conclusions

In conclusion, the presented findings show that use of first-line, TDM-driven infliximab to treat aGVHD in children may result in better clinical outcomes and good tolerability, with a variable pattern of serum cytokines and drug clearance shown according to the timing of treatment and disease extension, respectively. 

These results further strengthen the important correlation between the onset of therapy, disease control, and cure. Indeed, inhibition of TNF-α has been suggested in all phases of aGVHD treatment, as prevention, part of primary treatment, and, most commonly, as treatment for steroid-refractory or steroid-dependent aGVHD.

Future multi-center randomized controlled trials may verify our preliminary observations through use of infliximab as a part of the primary treatment for intestinal and liver aGVHD.

## Figures and Tables

**Table 1:** Clinical characteristics of patients with aGVHD treated using infliximab.

CHARACTERISTICS	Early Infliximab Group	Standard Infliximab Group	*p*-Value
Infliximab-concomitant therapy, number (%):			
Tacrolimus	13 (93)	14 (100)	0.847
MMF	1 (7)	2 (14)	0.540
Steroids	10 (71)	11 (78)	0.827
Ruxolitinib	3 (21)	0	<0.001 *
Patients, number (%)	14 (50)	14 (50)	1.000
Sex (male/female), number (%)	8 (57)/6 (43)	8 (57)/6 (43)	1.000
Age at treatment, years, median (range)	6.5 (0.6–17)	8 (1.1–17)	0.984
Body mass index, kg/m^2^, median (range)	17.36 (13.43–26.46)	17.03 (13.24–27.97)	0.222
Primary disease, number (%):			
Acute lymphoblastic leukemia	3 (21)	8 (57)	0.091
Acute myeloid leukemia	4 (29)	1 (7)	0.094
Myelodysplastic syndromes	4 (29)	2 (14)	0.386
Non-malignant	3 (21)	3 (22)	1.000
Transplant type, number (%):			
HLA-identical sibling	4 (29)	1 (7)	0.094
Related haploidentical	4 (29)	3 (21)	0.703
Matched unrelated	6 (42)	10 (72)	0.302
Graft type, number (%):			
Bone marrow	8 (57)	1 (7)	<0.001 *
Peripheral blood	6 (43)	13 (93)	0.084
Follow-up in survivors, months, median (range)	3.75 (2.1–59)	17.7 (7–35.8)	0.004 *
Interval HSCT to aGVHD onset:			
Median (range), days	22.5 (9–93)	15 (9–220)	0.984
>day + 100, number	0	1	<0.001 *
Maximum aGVHD grade, number (%):			
1	2 (14)	0	<0.001 *
2	10 (72)	4 (28)	0.076
3 or 4	2 (14)	10 (72)	0.002 *
Total number of organs involved, number (%):			
1	8 (58)	2 (14)	0.018 *
2	5 (35)	8 (58)	0.392
3	1 (7)	4 (28)	0.094
Skin stage, number (%):			
0–1	1 (50)	0	<0.001 *
2	1 (50)	6 (67)	0.007 *
3–4	0	3 (33)	<0.001 *
Liver stage, number (%):			
0–1	2 (29)	0	<0.001 *
2	5 (71)	6 (43)	0.762
3–4	0	8 (57)	<0.001 *
GI tract, number (%):			
0–1	2 (17)	0	<0.001 *
2	8 (66)	3 (43)	0.091
3–4	2 (17)	4 (57)	0.386
Other	3 (21)	6 (43)	0.289

aGVHD—acute graft-versus-host disease; HSCT—hematopoietic stem cell transplantation; GI—gastrointestinal; MMF—mycophenolate mofetil; * statistically significant values.

**Table 2:** Results of fixed and random effects of the final model.

	Value	SE	RSE (%)
Fixed effects			
V_pop_ (L)	3.433	0.336	9.79
CL_pop_ (L/day)	0.146	0.014	9.26
Standard deviation of the random effects			
IIV_V_	0.375	0.073	19.4
IIV_CL_	0.363	0.090	24.6
IOV_CL_	0.278	0.050	17.9
Residual variability			
Residual proportional error	0.316	0.018	5.65

SE—standard error; RSE—relative standard error; V_pop_ and CL_pop_—volume of distribution and clearance of patients’ population; IIV_V_ and IIV_CL_—interindividual variability in volumes of distribution and clearance, respectively; IOV_CL_—interoccasion variability in clearance.

## Data Availability

The data underlying this study will be shared on request to the corresponding author.

## Supplementary Materials

The following supporting information can be downloaded via the following link: https://www.mdpi.com/article/10.3390/cancers15143605/s1, Table S1: Cytokines values in early and standard condition. Caption. Table S2: Cytokine profile of healthy pediatric patients.
