# Supplementary material for: Advantage of First-Line Therapeutic Drug Monitoring-Driven Use of Infliximab for Treating Acute Intestinal and Liver GVHD in Children: A Prospective, Single-Center Study
Source: Cancers (Basel). 2023 Jul 13;15(14):3605. doi: 10.3390/cancers15143605 (PMC10376946; doi:10.3390/cancers15143605)
Supplement: Supplementary file 1 [file cancers-15-03605-s001.zip › cancers-2449790-supplementary.pdf]

Supplementary Table S1. Cytokines values in early and standard condition.

	Pre-infliximab		1-7 days		8-21 days		22-40 days		41-80 days		>81 days	
	**Early**	**Standard**	**Early**	**Standard**	**Early**	**Standard**	**Early**	**Standard**	**Early**	**Standard**	**Early**	**Standard**
**b-FGF**	17,68±2,59	23,44±2,75	21,92±1,29	19,44±1,42	21,41±1,82	17,45±1,77	21,61±1,68	20,94±2,00	23,12±1,63	20,8±1,54	28,73±1,37	22,28±2,54
**Eotaxin**	82,33±32,39	67,74±27,08	89,17±20,55	67,32±21,28	88,43±24,21	115,80±36,96	67,84±18,59	123,40±32,33	60,52±15,86	112,80±47,99	46,36±3,62	34,60±3,83
**G-CSF**	20,68±3,63	26,19±5,06	30,31±4,03	39,14±7,26	26,37±3,14	28,11±3,40	24,27±3,44	22,53±3,14	24,48±2,87	30,50±4,76	26,98±6,59	27,98±3,02
**IFN-γ**	122,10±26,18	152,50±19,47	126,20±8,45	108,40±10,65	139,30±12,59	109,40±9,15	136,90±15,96	181,50±30,48	126,20±11,41	160,60±20,23	122,10±20,06	109,90±21,47
**IL-1β**	7,59±1,10	8,60±1,59	16,36±4,06	13,70±2,48	11,69±2,03	9,32±2,22	9,73±1,82	11,20±2,62	11,74±2,21	12,11±2,19	12,12±4,79	10,53±2,98
**IL-1ra**	743,30±68,29	836,00±66,48	597,20±57,20	839,50±125,60	623,10±47,49	644,60±46,27	637,70±71,23	660,80±54,20	657,90±50,98	769,10±75,21	705,60±63,42	605,90±60,56
**IL-2**	24,81±1,61	27,97±2,52	34,89±2,93	34,01±2,25	29,57±2,34	26,07±1,54	31,38±2,05	29,31±2,70	30,11±3,18	28,76±1,59	35,51±6,95	32,42±5,05
**IL-4**	22,89±3,01	21,29±3,18	19,86±2,39	15,17±1,31	22,41±2,14	22,53±1,99	19,04±2,22	26,48±1,86	19,87±2,20	22,62±2,19	20,15±4,33	21,49±1,27
**IL-6**	71,52±12,85	98,02±14,60	59,86±17,34	71,06±11,91	44,61±7,14	66,20±9,62	80,59±17,33	87,42±22,45	56,15±9,64	75,46±20,08	53,49±6,03	64,15±7,65
**IL-7**	19,36±2,63	17,65±3,21	14,70±1,88	9,14±1,30	18,14±2,62	11,71±2,13	15,55±2,57	16,59±2,83	14,47±2,37	18,03±3,13	17,12±5,85	10,40±2,63
**IL-8**	28,69±1,79	27,45±2,44	30,47±1,81	28,36±1,39	28,87±1,92	28,04±2,30	28,26±2,63	26,27±2,73	27,25±2,29	29,28±1,39	27,83±2,21	29,20±3,03
**IL-9**	12,01±2,23	12,56±2,10	15,35±1,23	13,15±0,87	17,99±2,47	22,23±6,01	17,19±1,59	27,35±8,67	18,33±1,83	16,96±1,36	15,44±0,91	17,58±1,93
**IL12p70**	32,23±1,91	34,28±2,50	35,72±1,87	36,02±1,80	36,68±2,00	35,68±1,74	36,13±2,01	35,38±2,64	37,90±1,92	33,43±1,43	32,84±2,50	34,58±3,13
**IL13**	18,95±4,20	18,77±2,50	31,18±3,72	19,64±2,59	23,01±2,75	14,54±1,69	17,60±2,45	20,02±2,17	19,06±2,09	18,23±1,94	22,76±3,10	23,03±4,33
**IL17**	73,51±11,14	98,88±14,51	77,93±6,31	64,58±4,03	92,56±9,42	80,68±9,85	69,15±7,21	97,77±11,56	77,44±9,75	90,68±10,39	95,47±21,43	74,89±11,62
**IP10**	556,90±69,85	570,40±57,53	595,80±61,97	613,50±42,82	485,40±31,13	574,80±38,33	621,70±56,88	625,30±50,56	564,30±39,60	573,00±38,40	446,60±68,54	785,80±92,71
**MCP1**	75,89±13,31	106,60±16,83	78,96±9,61	108,30±18,25	63,49±6,33	90,15±11,78	98,85±11,97	82,24±7,76	93,09±8,15	89,34±10,24	67,65±4,81	76,01±17,15
**MIP1α**	7,11±0,53	8,25±1,63	6,08±0,64	4,75±0,50	5,79±0,63	4,91±0,67	4,98±0,60	7,25±0,87	5,60±0,73	5,69±0,44	5,46±0,77	5,17±0,63
**MIP1β**	63,19±12,78	57,99±14,18	74,02±13,83	38,71±5,31	60,96±8,05	54,55±10,19	55,72±9,32	40,50±5,80	55,71±9,86	54,59±8,45	39,49±4,28	116,40±31,78
**PDGF bb**	455,90±127,20	745,90±204,00	673,70±162,70	760,80±115,40	767,60±159,20	930,70±209,20	694,10±231,50	1045,00±209,30	651,80±163,30	707,50±88,20	1038,00±248,40	875,00±338,40
**TNF-α**	22,15±2,64	22,98±2,97	28,27±1,33	21,21±1,39	26,26±1,60	26,36±1,81	27,37±1,61	30,82±2,05	27,70±1,47	28,01±1,57	33,48±2,82	27,13±3,66

Mean±SE

Supplementary Table S2. Cytokine profile of healthy pediatric patients.

	**Healthy Control Values**
**b-FGF**	33,14±7,403
**Eotaxin**	52,02±3,603
**G-CSF**	13,70±2,556
**IFN-γ**	71,96±9,744
**IL-1β**	4,667±1,344
**IL-1ra**	140,5±7.398
**IL-2**	5,031±0.672
**IL-4**	1,880±0,231
**IL-6**	6,004±0.812
**IL-7**	5,688±0,619
**IL-8**	10,29±1.995
**IL-9**	14,54±3.842
**IL12p70**	28,46±2.096
**IL13**	5,889±1,078
**IL17**	41,36±10,68
**IP10**	163,4±6.549
**MCP1**	57,37±14.13
**MIP1α**	3,952±0,825
**MIP1β**	23,68±2,384
**PDGF bb**	253,4±44,40
**TNF-α**	13,47±1,367

Mean±SE
